# Healthcare Equipment and Personnel Reservoirs of Carbapenem-Resistant *Acinetobacter baumannii* Epidemic Clones in Intensive Care Units in a Tunisian Hospital

**DOI:** 10.3390/microorganisms11112637

**Published:** 2023-10-26

**Authors:** Sana Azaiez, Marisa Haenni, Asma Ben Cheikh, Mohamed Sahbi Chalbi, Aziza Messaoudi, Lamia Tilouch, Sana Bahri, Antoine Drapeau, Estelle Saras, Mariem Mtibâa, Rania Zouaoui, Houyem Said, Jean-Yves Madec, Agnese Lupo, Wejdene Mansour

**Affiliations:** 1 Laboratoire de Recherche Biophysique Métabolique et Pharmacologie Appliquée, LR12ES02, Faculté de Médecine Ibn Al Jazzar Sousse, Université de Sousse, Sousse 4002, Tunisia; sana.azaiez@famso.u-sousse.tn (S.A.); aziza.messaoudi@famso.u-sousse.tn (A.M.); sana.bahri@famso.u-sousse.tn (S.B.); mariem.mtibaa@famso.u-sousse.tn (M.M.); rania.zouaoui@famso.u-sousse.tn (R.Z.); 2 Unité Antibiorésistance et Virulence Bactériennes, ANSES—Université de Lyon, 69007 Lyon, France; marisa.haenni@anses.fr (M.H.); antoine.drapeau@anses.fr (A.D.); estelle.saras@anses.fr (E.S.); jean-yves.madec@anses.fr (J.-Y.M.); 3 Departement of Prevention and Security of Care, Sahloul University Hospital of Sousse, Sousse 4054, Tunisia; asma.bencheikh@famso.u-sousse.tn (A.B.C.); sahbi.chalbi.sc@rns.tn (M.S.C.); houyem.said@famso.u-sousse.tn (H.S.); 4 Faculté de Médecine Ibn Al Jazzar Sousse, Université de Sousse, Sousse 4002, Tunisia; 5 Laboratoire de Microbiologie Sahloul, University Hospital of Sousse, Sousse 4054, Tunisia; lamia.tilouch@fphm.u-monastir.tn; 6 Faculté de Pharmacie, Université de Monastir, Monastir 5019, Tunisia

**Keywords:** *bla*_OXA-23_, *bla*_NDM-1_, *armA*, AbRGI-1, Tn*6180*, Tn*2006*, Tn*125*, ST85, carbapenem-resistant *Acinetobacter baumannii*

## Abstract

Carbapenem-resistant *Acinetobacter baumannii* (CRAB) strains can cause severe and difficult-to-treat infections in patients with compromised general health. CRAB strains disseminate rapidly in nosocomial settings by patient-to-patient contact, through medical devices and inanimate reservoirs. The occurrence of CRAB in patients residing in the intensive care units (ICUs) of the Sahloul University hospital in Sousse, Tunisia is high. The objective of the current study was to determine whether the surfaces of items present in five ICU wards and the medical personnel there operating could serve as reservoirs for CRAB strains. Furthermore, CRAB isolates from patients residing in the ICUs during the sampling campaign were analyzed for genome comparison with isolates from the ICUs environment. Overall, 206 items were screened for CRAB presence and 27 (14%) were contaminated with a CRAB isolate. The items were located in several areas of three ICUs. Eight of the 54 (15%) screened people working in the wards were colonized by CRAB on the hands. Patients residing in the ICUs were infected with CRAB strains sharing extensive genomic similarity with strains recovered in the nosocomial environment. The strains belonged to three sub-clades of the internationally disseminated clone (ST2). A clone emerging in the Mediterranean basin (ST85) was detected as well. The strains were OXA-23 or NDM-1 producers and were also pan-aminoglycoside resistant due to the presence of the *armA* gene. Hygiene measures are urgent to be implemented in the Sahloul hospital to avoid further spread of difficult-to-treat CRAB strains and preserve health of patients and personnel operating in the ICU wards.

## 1. Introduction

*Acinetobacter baumannii* is a dreaded opportunist pathogen responsible for severe and invasive infections especially in patients with compromised health status and impaired immune system [1]. A problematic medical issue is the propensity of *A*. *baumannii* to develop multidrug resistance [2]. In particular, the dissemination of carbapenem resistance, mostly associated with acquisition of genes encoding beta-lactamases hydrolyzing carbapenems such as *bla*_OXA-23_ and *bla*_NDM-1_, limits the therapeutic options to treat severely ill patients [3,4]. Furthermore, certain carbapenem-resistant *A*. *baumannii* (CRAB) clones are associated with high mortality [5]. For all these reasons, *A*. *baumannii* belongs to the ESKAPE pathogen group (*Enterococcus faecium*, *Staphylococcus aureus*, *Klebsiella pneumoniae*, *Acinetobacter baumannii*, *Pseudomonas aeruginosa*, and *Enterobacter* species) [6] and in particular to the group for which the discovery of new therapeutic options is critically needed [7].

The majority of CRAB strains belong to internationally disseminated clonal complexes (IC). So far, nine internationally disseminated IC have been designed [8], but IC2 is by far the most prevalent [9], also in countries of the Mediterranean basin [10,11,12,13].

CRABs are mostly reported from nosocomial-acquired infections. Indeed, indwelling devices serve as vehicles for *A*. *baumannii* to the patients [14,15,16,17,18]. In turn, the contamination of indwelling devices can occur through the hands of medical care-giving workers [19,20]. Furthermore, surfaces of the hospital environment and medical material or equipment can serve as persistent reservoirs of *A*. *baumannii* [19,21]. Strains residing on these supports can thus be responsible for the colonization of several patients in the same ward, generating outbreaks [22,23,24].

The ability of *A*. *baumannii* to persist in the hospital environment is often attributed to adaptive features such as resistance to disinfectants [25,26], production of biofilm [27], surviving to desiccation [28,29,30], and developing antibiotic resistance [25,31]. Although the ability to resist to desiccation is often mentioned as a factor facilitating *A*. *baumannii* dissemination in hospitals, this adaptive feature has been observed in both epidemic and sporadic clones [29]. On the contrary, multidrug resistance is a distinctive feature of epidemic clones compared to sporadic ones [29].

Environmental contamination is estimated to contribute to up to 20% of all hospital acquired infections [32]. The World Health Organization’s guideline recommends a set of measures to prevent and control infections caused by CRAB in hospital settings. These measures include surveillance cultures and specific behavioral interventions such as hand hygiene, contact precautions, CRAB-colonized patient isolation (single-room isolation or cohorting) and environmental cleaning [33].

The objective of the present work was to investigate the role of medical equipment, furniture, personnel operating in the hospital, and patients in the dissemination of CRAB clones in a Tunisian hospital during a two-month time-lapse. 

The relatedness of the detected CRAB isolates was investigated at the genome level, using next-generation sequencing technologies. The presence of resistance genes and related genetic elements was characterized. This study provides an in-depth molecular investigation of CRAB isolates and stresses the necessity of hygienic measures in controlling pathogens dissemination in nosocomial environments.

## 2. Materials and Methods

### 2.1. Sampling and Wards

A cross-sectional study was conducted during October and November 2020, in the five intensive care units of the 690-bed Sahloul University hospital, in Sousse, Tunisia.

The Sahloul University hospital hosts 626 beds for seven hygiene control personnel (one hygienist for 100 patients). Cleaning procedures consist in the disinfection of floors and upper surfaces by quaternary ammonium compound (DDN-surf) twice a day, once in the morning and once in the evening. In intensive care units (ICUs, n = 5), 58 nurses and medical personnel operate for 29 patient beds. The ICUs are located on three floors (Figure S1) and the distribution of beds is as follows: ten for the general surgery (ICU-S), eight for cardiology (ICU-C), four for medical reanimation (M-ICU), four for cardio-vascular and thoracic surgery (CVTS) and three for pediatric intensive care unit (ICU-P). In each ICU, patient beds are separated by sliding curtains. During 2020, medical personnel operating in the ICUs provided care also in other wards. This was due to the COVID-19 crisis, which led to instantaneous changes in staffing requirements.

**Figure 1:**
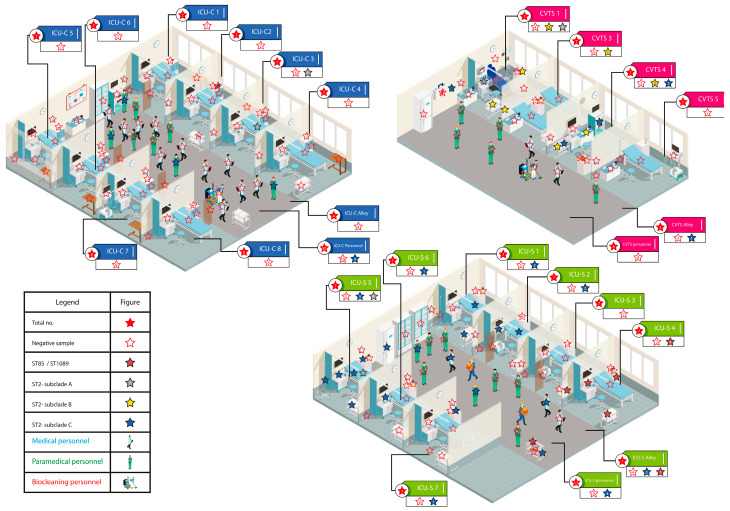
Graphic representation of three ICUs of the Sahloul University hospital with items or operating personnel colonized with carbapenem-resistant *Acinetobacter baumannii* strains.

Samples were collected by swabbing items and medical equipment with sterile swabs (eSwab, Copan, Murrieta, CA, USA). The items were located in areas of five ICU wards located on three different floors of the hospital (Figure 1). The wards included (i) cardiology (ICU-C) (n = 80, 60 environmental and 20 hand-swabs of hospital personnel); (ii) cardio-vascular and thoracic surgery (CVTS) (n = 40, 32 environmental and eight personnel samples); (iii) general surgery (ICU-S) (n = 69, 56 environmental and 13 personnel samples); (iv) pediatric (ICU-P) (n = 34, 26 environmental and eight personnel samples); and (v) medical (M-ICU) (n = 37, 32 environmental and five personnel samples) (Figure 1 and Table S1).

In particular, environmental samples focused on surfaces exposed to both patients and healthcare professionals such as door handles, patient beds, stethoscopes, liquid soap dispensers, medical devices used for reanimation, and syringe drivers, among others (Table S1). Hand swabs were taken from two hands of healthcare workers (medical doctors, nurses, technicians, trainees and cleaning staff). Eighteen clinical *A. baumannii* strains from patients hospitalized during the same period of the sampling campaign were provided by the Sahloul hospital bacteriology laboratory and were included in the analysis. These strains were isolated from patients residing in ICU-S, CVTS and other wards (Table 1). The study was approved by an ethical committee under the protocol HS-27-2023.

### 2.2. Bacteria Cultivation

Swabs were cultured for 24 h in Brain Heart Infusion sterile broth (Oxoid, Basingtoke, UK). From the culture, 10 µL were seeded onto MacConkey agar (Sigma, St. Louis, MI, USA) supplemented with imipenem (2 µg/mL). After a 24 h incubation at 37 °C, one colony for each observed morphology was sub-cultured and identified by biochemical profiles (Api20NE gallery, bioMérieux, Marcy-l’Etoile, France).

### 2.3. Antimicrobial Susceptibility Testing

Susceptibility to carbapenems (imipenem and meropenem), tigecycline, and amikacin was evaluated by strip gradient (E-test, bioMérieux, Marcy-l’Etoile, France) according to manufacturer’s instructions. Susceptibility to colistin was determined by broth microdilution according to CLSI/EUCAST recommendations [34]. Further antibiotics (ticarcillin (75 µg), ticarcillin/clavulanic acid (75 µg/10 µg), piperacillin (100 µg), piperacillin–tazobactam (100 µg/10 µg), ceftazidime (30 µg), cefepime (30 µg), gentamicin (10U.I), ciprofloxacin (5 µg), and tobramycin (10 µg)) were tested by disc diffusion. Strains ATCC 25922 and ATCC 27853 were used as quality controls. The inhibition diameter was measured and recorded using the automated system Scan 4000 (Interscience, St. Nom-la-Bretèche, France). Susceptibility categorization was based on epidemiological cut-offs provided by EUCAST (mic.eucast.org) or breakpoints available from CA-SFM.

### 2.4. Whole-Genome Sequencing and Bioinformatics Analysis

The genomes of all *Acinetobacter baumannii* strains (n = 55) were sequenced. DNA was extracted using the NucleoSpin microbial DNA extraction kit (Macherey-Nagel, Hoerdt, France). Libraries were prepared using Nextera XT technology and sequencing was performed on a NovaSeq 6000 instrument (Illumina, San Diego, CA, USA), generating short-reads (100 bp). Reads were qualitatively sorted and assembled de novo using Shovill 1.0.0 (Table S1). The quality of the assemblies was assessed using QUAST v4.5. Sequence type and resistance genes were determined using the Center Genomic Epidemiology (https://www.genomicepidemiology.org/, accessed on 21 July 2023) online tools MLST Finder 2.0.4 and ResFinder 3.2. The presence of genes related to adhesion was examined using the Virulence Factors Data Base (http://www.mgc.ac.cn/, accessed on 9 October 2023) or by blast using as reference the locus *csuE* (MJHA01000005.1, region 1049251-1048232).

For three strains (58429, 58450 and 58477), the genome was sequenced generating long-reads, as well. The Oxford Nanopore technology was used (Nanopore, MinIon, Oxford, UK) after library preparation (SQK-NBD112-24) and using the r10.4.1 flow cell. For these isolates, long and short reads were hybrid assembled using Unicycler v.0.4.8.

Annotation of the genomes was achieved using RASTtk available from the BV-BCR platform. Analysis of genetic elements carrying antibiotic resistance genes was carried out using blastn 2.14.0, and the Easyfig 2.2.5 win was used for representation and alignment.

Genomic relatedness of *A. baumannii* strains was evaluated by PyMLST v.2.0.1 using the *A*. *baumannii* cgMLST database [35] which currently includes 2390 alleles. After allele sequence alignment (MAFFT v.7), a phylogenetic tree was constructed on the matrix of distance using the Neighbor join algorithm. Annotation and representation of the tree was achieved using iTOL v.6.

## 3. Results

### 3.1. CRAB Prevalence in ICUs Wards

In total, 27 out of 206 samples (14%) from ICUs items were positive for CRAB colonization. Two samples generated two morphologically distinct colonies, thus 29 CRABs from equipment and hospital items were analyzed (Table S1). Positive samples were detected in the ICU-C, CVTS and ICU-S wards, while none were detected in the Ped-CVTS and the M-ICU wards.

In the ICU-C, only one sample (1/60) was CRAB positive and detected in one patient’s bed. A total of four people out of 20 operating in the ICU-C were CRAB-colonized on the hands. In the ward, seven CRAB-infected patients were residing (Figure 1 and Table S1).

In the CVTS ward, 9/32 screened objects were CRAB positive, while one positive patient was present in the ward. No positive personnel were found in this ICU. CRAB strains were found on three care carts, and one each from a defibrillator, a dial device, a soap dispenser, a hands-disinfectant dispenser, and an electrocardiogram device. The items colonized by CRAB isolates were located in three different areas and the hallway (Figure 1 and Table S1).

The highest contamination was detected in the ICU-S, where 18/56 positive samples were found containing 19 distinct CRAB isolates. Care carts and patient beds were the most contaminated surfaces (four of each of those were positive), followed by stethoscopes (n = 3) and syringe drivers (n = 2). A single defibrillator, intravenous pole, respiratory assistance device, soap dispenser, the handle of a refrigerator, and the surface of a furniture were found positive for CRAB presence. These items were located in different areas of the same ward and the hallway. In the ICU-S, 4/13 people belonging to medical and paramedical personnel were CRAB positive (Figure 1 and Table S1).

In the year of the sampling campaign for the current study (2020), 195 *A. baumannii* isolates were collected from all wards of the Sahloul University hospital and were collected in the diagnostic laboratory of the hospital. The majority of these isolates (188/195, 96.5%) were resistant to imipenem and ceftazidime and were isolated (n = 153) from patients residing in different ICUs, whereas the remaining 35 strains were isolated from other hospital wards. The sex ratio was 3:1, with women accounting for 1/3 of the strains isolated (63/188) and men for 125/188 strains. Eighteen out of the collected 188 CRAB isolates, coming from patients hosted in the investigated ICUs contemporaneously to the sampling campaign, were included in the study for comparative analysis with isolates collected from the hospital environment. Most of these isolates (13/18) were found in infected male patients. For 14 patients, age was known and ranged from 36 to 78 years, with a median of 58 years. The majority of patients was previously hosted in another ward (Table 1 and Table S1). Six out of the twelve patients for which the outcome data are available died at the hospital.

### 3.2. PyMLST of CRAB Isolates from Hospital Environment, Personnel and Patients

In total, 55 CRAB strains were characterized, including 29 strains from ICUs items, eight strains from colonized hands of eight people working in the ICUs, and 18 strains from 18 patients, including one patient from M-ICU where no CRAB-colonized items were detected.

**Figure 2:**
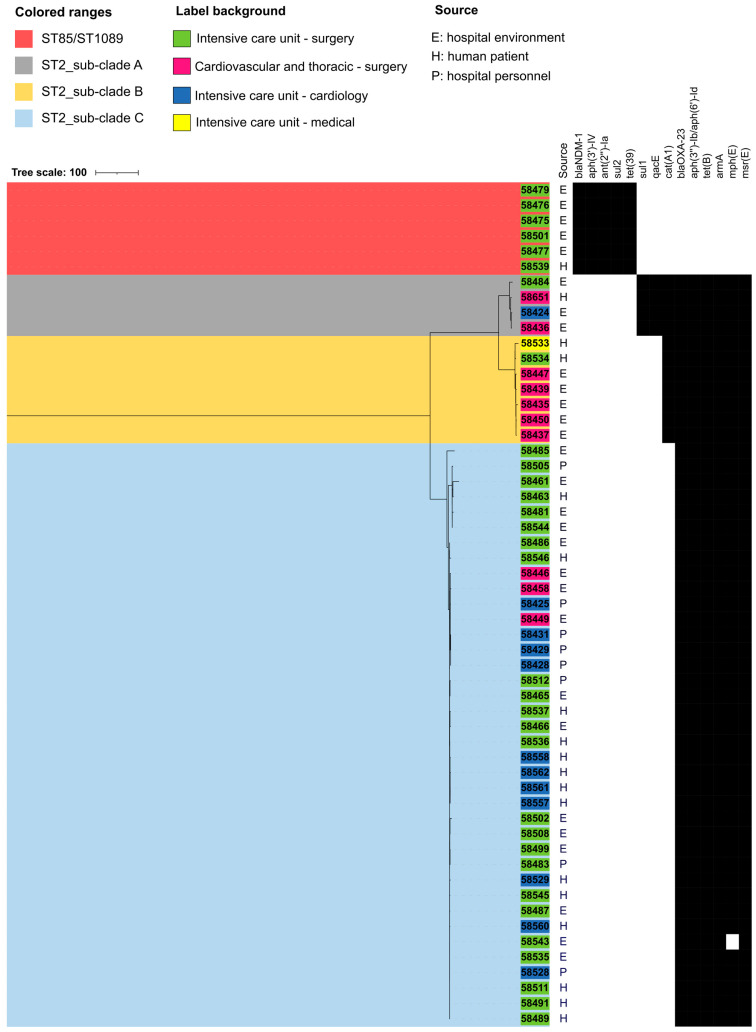
cgMLST-based phylogeny of the 55 CRAB isolates.

The sequence type (ST) of the CRAB strains was assigned according to the two available MLST schemes. The Institute Pasteur scheme assigned two STs: ST85 (corresponding to ST1089 of the Oxford scheme) to six isolates and ST2 to all the remaining 49 isolates. Among the ST2 isolates, the Oxford scheme discerned three allelic profiles (Table S1). In agreement with the Oxford typing, the pyMLST analysis evidenced the presence of four clades among the 55 CRAB genomes (Figure 2). Isolates belonging to ST85/ST1089 grouped in a unique clade and were isolated from one patient and items located in area 4 of the ICU-S as well as in the contiguous hallway (Figure 1 and Figure 2). Among the ST2 strains, four clustered in the sub-clade A (Figure 2). These isolates were on items of three ICU wards and one patient (Figure 1 and Figure 2). The sub-clade B included seven isolates that were recovered on surfaces of items in the CVTS and two patients residing in other wards (ICU-M and ICU-S) (Figure 1 and Figure 2). The remaining 38 isolates constituted the largest sub-clade, named sub-clade C, and were recovered from the hands of personnel, medical equipment and causing infection in human patients. This clone, in addition to being the most common, disseminated in three different wards (Figure 1 and Figure 2).

### 3.3. Antimicrobial Susceptibility of CRAB Strains and Antibiotic Resistance Genes

Carbapenemase-producing isolates were confirmed to be resistant to meropenem and imipenem. Similarly, the CRAB strains were co-resistant or had intermediate susceptibility to other beta-lactams (Table S1). A high proportion of isolates presented non-susceptibility to tigecycline (84%) and to amikacin (89%). All isolates were colistine susceptible but ciprofloxacin resistant. Isolates were in silico cefiderocol-susceptible (absence of major contributors to cefiderocol resistance: *bla*_PER-type_ [36] and *bla*_NDM-9_ [37]). All but one isolate carried the macrolide resistance genes *msr*(E) and *mph*(E). Further resistance genes occurred with a pattern reflecting the phylogenetic grouping.

CRAB strains belonging to ST85/ST1089 carried a metallo-carbapenemase encoding gene *bla*_NDM-1_ and the *aph*(3′)-*VI* gene, usually associated to amikacin resistance [38]. However, strains carrying the *aph*(3′)-*VI* gene of this study presented MIC values ranging from 2 to 6 mg/L, thus corresponding to a wild-type phenotype. Analysis of the sequence upstream the transcription initiation codon (using bprom [39]) predicted the presence of −35 (CTCTCT) and −10 (GTTTTTAA) boxes, suggesting the presence of a promoter region and potential for the gene expression and amikacin resistance. Furthermore, ST85/ST1089 strains also carried the *ant*(2″)-*Ia* gene conferring resistance to gentamicin and tobramycin [40] against which the strains of this study had intermediate susceptibility. Finally, ST85/ST1089 strains carried the sulfonamides resistance gene *sul2* and the tetracycline resistance determinant *tet*(39) (Table S1).

All ST2 CRAB strains were OXA-23 producers and harbored the ribosome–methylase encoding gene *armA* (Figure 2, Table S1). Strains included in the ST2 sub-clades carried accessory resistance genes according to the phylogenetic grouping. For instance, strains ST2 sub-clade A carried a chloramphenicol-resistance gene *catA1*, the *sul1* gene conferring sulfonamide resistance and the quaternary ammonium resistance gene *qacE* (Table S1). The gene *catA1* was also present in the strains of the ST2 sub-clade B that also contained the *tetB* gene (Table S1). Strains of the sub-clade C were devoid of the *sul1*, *qacE* and *catA1* genes (Table S1).

A blastn search revealed that all ST2 and ST85 strains carried the *gspO* gene, encoding a type four pilus involved in host cell adhesion [41], with a 100% and a 98% nucleotide identity, respectively, with the locus ACICU_RS01815 from the ACICU strain. The gene *csuE*, mediating adhesion to abiotic surfaces [42], was present in all isolates with 97–98% nucleotide identity with the *csuE* locus of strain ATCC19606. 

### 3.4. Genetic Elements Carrying Acquired Antibiotic Resistance Genes

The full-assembled genomes were obtained for representative isolates such as #58477 belonging to ST85, #58450 belonging to the ST2 sub-clade B and #58429 belonging to the ST2 sub-clade C. Contigs obtained for strain #58651 belonging to the ST2 sub-clade A were used for mapping on nucleotide sequences of complete genetic elements.

**Figure 3:**
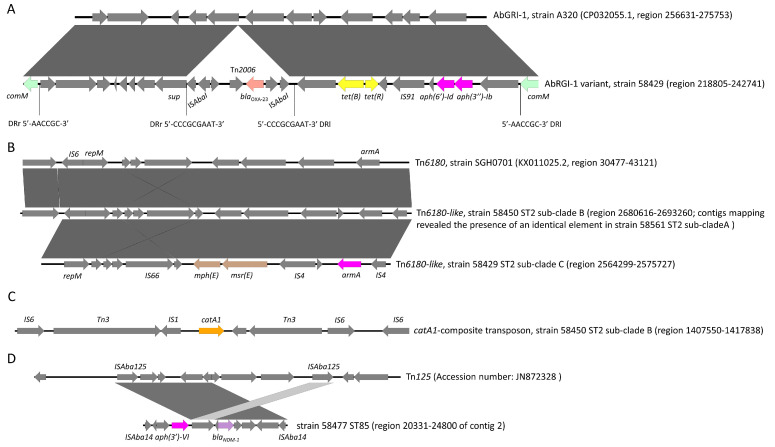
Schematic representation of genetic elements carrying *bla*_OXA-23_ (**A**) and *bla*_NDM-1_ (**D**) carbapenemase-encoding genes and the pan-amioglycosides resistance *armA* (**B**) and blast alignment with similar genetic elements from the NCBI database (Easyfig 2.2.5 win). In (**C**) graphical representation of the genetic element carrying the *catA1* gene.

Strain #58429 (ST2 sub-clade C) carried the *bla*_OXA-23_ gene on a Tn*2006* transposon [43] flanked by nine nucleotide direct repeat sequences (5′-CCCGCGAAT-3′), inserted in the *sup* gene of an AbGRI-1 island variant [44]. The AbGRI-1 island also carried the tetracycline resistance genes *tet*(B)/*tet*(R) and streptomycin resistance genes *aph*(6′)-*Id*-*aph*(3′’)-*Ib*. The AbGRI-1 island, flanked by six nucleotide direct repeat sequences (5′-AACCGC-3′), was, in turn, inserted in the *comM* locus at 841 nucleotides from the initiation codon (Figure 3A).

Strain #58450 (ST2 sub-clade B) carried an AbGRI-1 variant identical to that characterized in strain #58429 (ST2 sub-clade C). Also in strain #58450, the AbGRI-1 island was inserted into the *comM* locus (100% coverage and 100% nucleotide identity, region 3889214-3865278 of the genome). Furthermore, strain #58450 carried a second copy of *bla*_OXA-23_-Tn*2006,* which was inserted in a locus predicted to encode a xanthine dehydrogenase maturation factor (XdhC) at 716 nucleotides from the initiation codon. This copy of Tn*2006* transposon was flanked by nine nucleotide direct repeat sequences (5′-AGTTTTAAT-3′).

Mapping contigs of #58651 (ST2 sub-clade A) against the nucleotide sequence of the AbGRI-1 island suggested that this element was present in strain 58651, as well.

Strains belonging to ST2 sub-clades carried the ribosomal methylase encoding gene *armA* embedded between two copies of IS*4*-type insertion sequences. In turn, the IS*4*-*armA*-IS*4* element was part of a partial Tn*6180* transposon*,* named also AbGRI-3 island [45]. Representative strains belonging to ST2 sub-clades A (58561) and B (58450) carried an identical Tn*6180*-like transposon, whereas strain #58429 of the ST2 sub-clade C lacked a locus encoding for the transposase of a IS*6* insertion sequence (Figure 3B). In all the above-mentioned ST2 strains, the Tn*6180*-like transposon was inserted in the chromosome between a gene encoding a MFS-type transporter and a gene encoding a putative lipoprotein. No direct nucleotide sequence repeats could be observed.

ST2 sub-clades A and B harbored also a *catA1* gene (Figure 2 and Table S1). In strain #58450 (ST2 sub-clade B), the *catA1* gene was located on a *Tn3* transposon, surrounded by two copies of IS*6* insertion sequences (Figure 3C). This composite transposon was inserted into the chromosome in a *nicP_1* gene, encoding a porin-like protein, causing its partial deletion (at nucleotide 270 from the initiation codon). Similarity searches in the NCBI database evidenced that similar composite transposons (100% coverage with at least 99.99% nucleotide identity) were present in other *A*. *baumannii* ST2 strains, such as A320, ST2 (CP032055.1), but also in strains belonging to other sequence types like LUH6011, ST46 (CP031383.1) and in strains of the Enterobacterales family (*Serratia marcescens* strain SCH909, CP063238.1). A mapping of contigs of #58651 strain (ST2 sub-clade A) suggested the presence of the *catA1* element (99% coverage) at 99.99% of nucleotide identity.

Strain #58477 (ST85/ST1089) carried the *bla*_NDM-1_ and *aph*(3′)-*VI* genes on a transposon bounded by two copies of IS*Aba14* and showing similarities with Tn*125* [46] (Figure 3D). A similarity search in the NCBI database displayed that the IS*Aba14*-embedded element (100% coverage and 100% nucleotide identity) was present in several *Acinetobacter* spp. including *Acinetobacter nosocomialis* (CP045561.1), *Acinetobacter johnsonii* (CP043307.1), *Acinetobacter lwoffii* (CP059301.1), but also in Enterobacterales members (*Escherichia coli*, AP018572.2; *Proteus mirabilis*, AP018566.2; *Klebsiella pneumoniae*, LR697132.1) with both chromosomal and plasmid localization. The Tn*125*-like element in strain #58477 was located on a 27004 nucleotide contig, including loci encoding components of a type 4 secretion system and a *rep* gene. The replicase shared a 97.75% amino acid identity with the Rep3-T27 type [47], suggesting the localization of the Tn*125*-like element on a plasmid. With the exception of the Tn*125*-like element, no significant similarities with other plasmids present in public repositories and carrying *bla*_NDM-1_ were observed.

Strains of the ST2 sub-clade A type harbored also *sul1* and the quaternary ammonium compound resistance gene *qacE* (Table S1). In strain #58651, these genes co-localized on a unique contig, associated to a transposase of the IS*6* family encoding gene.

## 4. Discussion

Fifteen years ago, Perez et al. [48] highlighted that, together with the impressive propensity to acquire and integrate antibiotic resistance genes, other remarkable features of *A*. *baumannii* are its ability to cause hospital-acquired infections, to generate outbreaks in hospitals and disseminate at the global scale. *A*. *baumannii* outbreaks can occur from a single source or from multiple sources [49]. Dissemination can start from a colonized patient and then transmission to other patients can occur by direct contact (skin or air droplets), through colonized hospital items, or through colonization of hands of hospital personnel [49]. The colonization of medical equipment and other items can serve as persistent reservoirs for continuous spread of the microorganism.

In Africa, hospital-acquired infection prevalence is estimated to be at 13% and *A*. *baumannii* is one of the contributing pathogens [50]. In particular, CRAB is endemic in the Mediterranean basin [51]. Polyclonal CRAB expansion has been observed in the Arabian league countries, with *bla*_OXA-23_ as the most prevalent carbapenemase-encoding gene, and with the emergence of *bla*_NDM-1_ [51].

In the Sahloul University hospital in Sousse, Tunisia, the first report of CRAB dates back to 2002 involving 20 patients suffering from different pathologies. Molecular typing of the isolates from the 20 patients highlighted the genetic relatedness of the isolates [52]. The 20 patients did not reside in the hospital contemporaneously, suggesting the presence of persistent reservoirs of CRAB strains in the hospital environment. The presence of CRAB in the Sahloul University hospital has been continuously reported [53]. 

In 2020, 188 CRAB strains were isolated in the microbiology diagnostic laboratory, representing 96.5% of the overall number of *A*. *baumannii* isolates (n = 195) detected in January and November 2020. This high occurrence of CRAB strains, in particular in patients residing in ICUs, prompted us to undertake an investigation for evaluating the contamination of items present in the ICU wards and the colonization of hospital personnel operating in those wards. Three out of the five investigated ICUs were positive for the presence of CRAB strains on the surface of items present in the areas of the wards. In pediatric and medical ICUs, no CRAB strains were found. The medical ICU principally hosted COVID-19 patients, probably explaining such lower incidence compared to the other wards. Overall, 31 distinct CRAB strains were found colonizing surfaces (beds, furniture, soap and disinfectant dispenser, refrigerator handle, care cart, n = 20) and medical equipment (defibrillator, electrocardiogram and dial device, stethoscope, syringe driver, respiratory assistance device, intravenous pole, n = 11). Also, medical personnel (n = 8/54 samples, 15%) operating in two ICUs, ICU-C and ICU-S, were colonized on the hands.

MLST of CRAB isolates from patients, hospital items and medical personnel evidenced that they belonged to two sequence types: the globally spread ST2 and ST85. Allelic variation analysis of the core genome highlighted that among CRAB ST2 strains, three sub-clades could be discerned (A, B and C), with sub-clade C as the most diffused. This analysis confirmed that CRAB strains causing infections in patients residing in the ICUs shared extensive genomic similarities with the strains found on the items and medical devices used in the ICUs wards where CRAB-infected patients were hosted, suggesting a shuffling of CRAB strains between inanimate hospital surfaces and patients. These data demonstrate a polyclonal dissemination of CRAB in the Sahloul University hospital. To extinguish such dissemination, the closure of wards is sometimes necessary [49], with consequent costs and impairment of public health services. CRAB strains from patients were sampled 48 h after their hospital admission, suggesting that these strains caused hospital-acquired infections. Actually, in the Sahloul University hospital, patients were not screened for CRAB colonization at admission, not excluding the fact that their CRAB colonization occurred before hospital admission, but no further information such as previous hospitalization or antibiotic therapies is known for these patients. More in general, screening of patients before hospital admission and implementation of special hygiene measures for CRAB-colonized patients could be a cost-effective method to prevent CRAB propagation in hospitals. The medical personnel that resulted positive for CRAB hands colonization operated in ICU-S and ICU-C and were colonized with ST2 sub-clade C, the most diffused clone among the ICU wards. This result suggests that, regardless of the way of entrance in the ICUs, CRAB dissemination was mainly facilitated by the colonization of medical personnel hands. Colonized hands, indeed, have been reported as the most effective method for CRAB strains dissemination in the outbreak context [49].

The endemic epidemiological situation of CRAB in Tunisia is corroborated by the occurrence of outbreaks in the hospitals of other cities of the country, for instance, in the “La Rabta University hospital” in Tunis [54,55], also involving a clone, ST158, typically disseminating in the Middle East Countries [56]. Another example was provided by the Great Burn Center in Ben Arous, in 2019 [57]. Here, a dedicated study to investigate the incidence of CRAB-caused infections suggested that no variations occurred at a significant level for the incidence or the prevalence of antibiotic resistance during the period 2012–2020. However, the rate of patient colonization by CRAB on the skin or in the central venous catheter was high (49.3%, n = 815) and represented a risk factor for infection development [58]. Other investigations, conducted on patients screened at the admission at the Charles Nicolle hospital, focusing on rectal carriage of CRAB, highlighted a lower colonization prevalence (4.8%, n = 63). However, 15% of patients became colonized with CRAB after 8 days from admission and 1/39 developed infection with a CRAB strain [59]. A molecular characterization of isolates from patients and CRAB isolated from hospital items revealed that patients and hospital items shared the same clones [60]. Unfortunately, molecular typing of the isolates of these studies was not carried out impeding to unveil possible inter-hospital dissemination of CRAB strains. Inter-hospital CRAB spread is likely to occur considering the fact that patients are frequently transferred among hospitals. For instance, in 2018, a ST85-NDM-1 *A*. *baumannii* producer was isolated in the university hospital of Monastir [61]. Sporadic ST85-NDM-1 *A*. *baumannii* producers (n = 8) from patients originating from North Africa, including Tunisia, have been previously described [62,63,64]. In our investigation, one patient was infected with a ST85-NDM-1 *A*. *baumannii* producer and this clone, although, limited to ICU-S ward, was able to colonize hospital item surfaces. Our findings offer warning regarding the persistence of this clone in Tunisia with potential inter-hospital dissemination.

In the ST85 strain, the *bla*_NDM-1_ gene together with gene *aph*(3′)-*VI* was located on a partial Tn*125* embedded by two copies of IS*Aba14*. This genetic asset has been previously reported in ST85 *A*. *baumannii* [63,64] and in isolates belonging to other sequence types [65]. The *aph*(3′)-*VI* gene is known to confer amikacin resistance; however, ST85 strains carrying the *aph*(3′)-*VI* gene retained amikacin susceptibility. Isolates from other analyses carrying the same genetic element presented a similar wild-type susceptibility [63,64]. Analysis of the sequence upstream the transcription initiation codon of *aph*(3′)-*VI* gene suggested the presence of −35 and −10 boxes that were spaced by 20 nucleotides. Whether this predicted promoter configuration could allow efficient transcription remains unknown. Further investigation of the expression of the *aph*(3′)-*VI* will be carried out.

CRAB strains of the current study carried further resistance genes, which were located on genetic elements reported from globally expanded clones, like AbGRI-1 that aggregated multiple resistance genes (*bla*_OXA-23_, *tet*(B), *aph*(3″)-*Ib* and *aph*(6)-*Id*) in a unique element. The Tn*6180* transposon, carrying a copy of IS*6*-like insertion sequence at the 5′ side, carried the *armA* gene together with the macrolide resistance genes *mph*(E) and *msr*(E). This element was identical in strains belonging to ST2 sub-clades A and B, whereas those of the ST2 sub-clade C lacked the IS*6*-like insertion sequence. This organization suggests a more recent diversification of the ST2 sub-clade C from the common ancestor with ST2 sub-clades A and B. Overall, the genetic elements characterized in the strains of this study are similar to those found in globally expanded clones and serve as reservoirs for further antibiotic resistance spread. Most of strains analyzed in this study were susceptible to colistin, an antibiotic problematic for toxicity and resistance selection in therapeutic implementation. It is frightening that CRAB strains are rapidly developing resistance to the most recent commercialized antibiotics, like cefiderocol [66,67] and the combination ceftazidime/avibactam [68]. Thus, preventing *A*. *baumannii* infections remains a necessary strategy to preserve the health of patients with risk factors for *A*. *baumannii* infection development.

## 5. Conclusions

The presence of CRAB strains belonging to globally disseminated clones or emerging ones is endemic in the Sahloul University hospital. The colonization of inanimate surfaces and medical personnel can nourish the circulation of these strains for an undetermined period. Cleaning and disinfection of the ICUs areas was performed in the morning of the sampling campaign day. Evidently, this procedure was not effective to eradicate the strains from the hospital environment, suggesting that, together with antibiotic resistance, disinfectant resistance could favor the persistence of these strains in the hospital environment. In addition, colonization of personnel hands could have been responsible for recontamination of surfaces even soon after cleaning. Because of multiple CRAB sources and polyclonal dissemination in a hospital environment, extraordinary hygienic measures might be necessary, including patient isolation, reinforced hand hygiene, cleaning and more efficacious disinfection. Infection prevention and hygiene are precious weapons to fight against this microorganism. 

## Figures and Tables

**Table 1:** Features of *A. baumanni* strains isolated from patients hospitalized in ICUs (October–November 2020).

Strain	Ward	Original Ward	Pathology	Treatment	Outcome
58546	ICU-S	ER	Head trauma	IMI, COL, VAN	Transferred
58534	ICU-S	-	-	-	-
58535	ICU-S	Surgery	Septic shock	IMI, AMI, FLUC	Died
58536	ICU-S	ER	Head/chest trauma	AMC, GEN	Transferred
58537	ICU-S	Surgery	Post-surgery sepsis	CTX, GEN, TAZ, AMI, MEM, VAN	Died
58539	ICU-S	-	-	-	-
58543	ICU-S	ER	Hemoperitoneum	TAZ, CIP, IMI, COL, VAN, AMC	Died
58544	ICU-S	ER	Pneumothorax	AMC	Dismissed
58545	ICU-S	ER	Polytrauma	IMI, COL, VAN	Dismissed
58557	ICU	COVID unit	ARD	IMI, COL, VAN	Dismissed
58558	ICU	ER	ARD	IMI, COL	Died
58560	ICU	Nabeul Hospital	ARD	IMI, COL	Died
58561	ICU	COVID unit	ARD	IMI, COL	Died
58562	ICU	COVID unit	ARD	COL, AZI	-
58651	CVTS	Cardiology	Surgical valve replacement	IMI, COL, AMI	Dismissed
58528	M-ICU	ER	ARD	TAZ, CIP, TEC	Dismissed
58529	M-ICU	-	-	-	-
58533	Orthopedics	-	-	-	-

ICU-S, Intensive care unit surgery; ICU, Intensive care unit; CVTS, Cardio-vascular and thoracic surgery; M-ICU, Medical Intensive care unit; ER, Emergency room; ARD, Acute respiratory distress; IMI, Imipenem; AMI, Amikacin, FLUC, Fluconazole; AMC, Amoxicillin + clavulanic acid; GEN, Gentamicin; CTX, Cefotaxime; TAZ, Tazobactam; CIP, Ciprofloxacin; MEM, Meropenem; VAN, Vancomycin; COL, Colistin; AZI, Azithromycin, TEC: Teicoplanin.

## Data Availability

The project was deposited in DDBJ/EMBL/GenBank under the BioProject accession number PRJNA999198.

## Supplementary Materials

The following supporting information can be downloaded at: https://www.mdpi.com/article/10.3390/microorganisms11112637/s1, Figure S1: Graphical representation of the Sahloul University hospital and location of Intensive Care Units. Table S1: Genomic features of *Acinetobacter baumannii* strains from ICUs items, hospital personnel and patients. Table S2: WGS quality controls.
