# Supplementary material for: Healthcare Equipment and Personnel Reservoirs of Carbapenem-Resistant Acinetobacter baumannii Epidemic Clones in Intensive Care Units in a Tunisian Hospital
Source: Microorganisms. 2023 Oct 26;11(11):2637. doi: 10.3390/microorganisms11112637 (PMC10672855; doi:10.3390/microorganisms11112637)
Supplement: Supplementary file 1 [file microorganisms-11-02637-s001.zip › Figure S1.pdf]

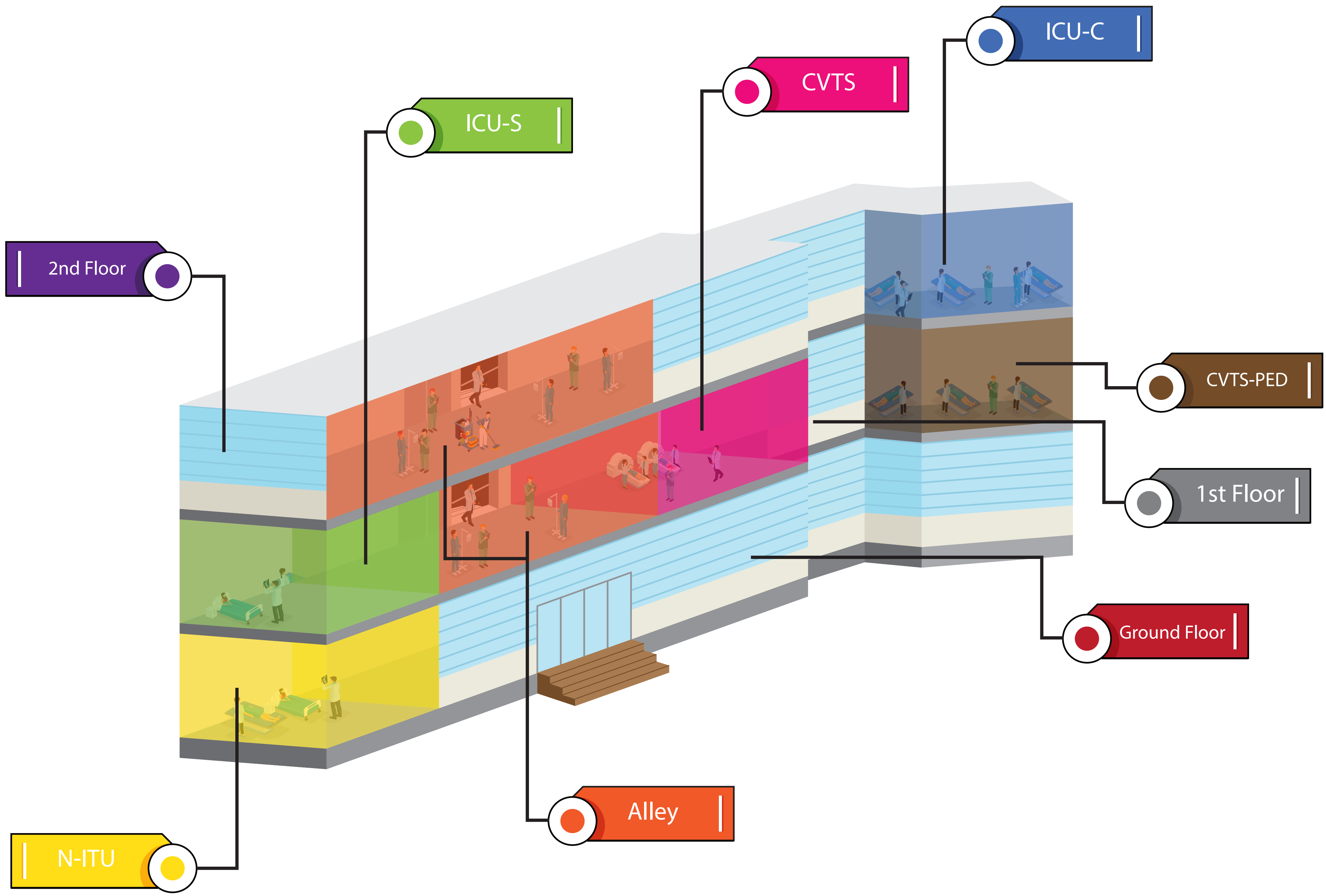


Figure S1. Graphical representation of the Intensive Care Units location in the Sahloul University hospital
